# The association between macronutrient intakes and coronavirus disease 2019 (COVID-19) in an Iranian population: applying a dynamical system model

**DOI:** 10.1186/s41043-023-00448-5

**Published:** 2023-10-26

**Authors:** Somayeh Ghiasi Hafezi, Najmeh Seifi, Hossein Bahari, Maryam Mohammadi, Atena Ghasemabadi, Gordon A. ferns, Ehsan Mosa Farkhani, Majid Ghayour-mobarhan

**Affiliations:** 1 International UNESCO Center for Health-Related Basic Sciences and Human Nutrition, Mashhad University of Medical Sciences, Mashhad, Iran; 2 Student Research Committee, Mashhad University of Medical Sciences, Mashhad, Iran; 3 Esfarayen University of Technology, Esfarayen, North Khorasan Iran; 4 Brighton & Sussex Medical School, Division of Medical Education, Falmer, Brighton, Sussex UK; 5 Deputy of Health, Mashhad University of Medical Sciences, Mashhad, Iran; 6 Metabolic Syndrome Research Center, Mashhad University of Medical Sciences, Mashhad, Iran

**Keywords:** Macronutrients, Nutrition, Diet, COVID-19, SARS-COV2, Dynamical system

## Abstract

**Aims:**

The possible role of lifestyle including diet on immunity led us to investigate the association between dietary macronutrient intake and COVID-19 in an Iranian population.

**Methods:**

Dietary intakes were recorded in the first phase of the MASHAD cohort study (started in 2007), using a 24-h dietary recall. To determine the COVID-19 incidence, data from all PCR-positive patients in Mashhad were recorded between February 2020 and June 2022. Dietary macronutrients were included in the regression model, adjusting for age and sex. System dynamical models were also applied.

**Results:**

The analysis included 1957 participants, including 193 COVID-19-positive patients. Dietary intakes of non-starch polysaccharides (NSP) and fiber were significantly lower in COVID-19 patients (*P* < 0.05). After adjusting for age and sex, starch and total sugar were significantly associated with COVID-19 infection ((OR = 1.0008, *P* = 0.001) and (OR = 1.0006, *P* = 0.026), respectively). There was also a significant association between dietary fiber intake and hospitalization (OR = 0.99, *P* = 0.018). In the dynamical system models, dietary intakes of cholesterol, polyunsaturated fatty acids (PUFA), and total sugar above 180.2 mg, 13.11 g, and 79.53 mg, respectively, were associated with an increased susceptibility to COVID-19 infection, while dietary fiber had a protective role.

**Conclusion:**

Dietary intake of starch and total sugars was associated with increased odds of COVID-19, while fiber intake decreased the odds of hospitalization due to COVID-19. The dynamical system models showed that dietary intake of cholesterol, PUFAs, and total sugar was associated with an increased risk of COVID-19, while fiber had a protective role.

## Introduction

Acute respiratory syndrome coronavirus-2 (SARS-CoV-2) was first reported in December 2019 in Wuhan, China. The extremely high transmission rate of this strain of SARS-CoV-2 led to the global pandemic of the coronavirus disease 2019, also known as COVID-19 [1]. Globally, as of 18 November 2022, there have been 633,601,048 confirmed cases of COVID-19, including 6,596,542 deaths, reported to WHO [2].

Characteristic clinical manifestations of COVID-19 include fever, dry cough, and fatigue, often with pulmonary involvement. In severe cases of the disease, rising levels of cytokines (IL-2, IL-7, IL-10), granulocyte colony-stimulating factor (G-CSF), monocyte chemotactic protein (MCP), and TNF-a have been observed. For this reason, the inflammatory cascade that causes this cytokine storm is a critical factor in the cause of the acute respiratory syndrome and extrapulmonary organ failure, which shows the importance of the immune system in developing this disease [3, 4]. Fighting new emerging viruses has always been a challenge. However, there is growing evidence for the essential role of nutrition in maintaining an optimum immunity against this pathogenic infection [3, 5]. According to the nutritional guidelines set by the World Health Organization for adults, eating a balanced diet rich in vitamins, minerals, dietary fiber, proteins, and antioxidants helps prevent disease [6].

Macronutrients, including proteins, carbohydrates, and fatty acids, supply the necessary energy for tissue growth and function [7]. Immune modulation by macronutrients has been studied in laboratory animals and human interventional studies testing the effect of their intake on immune outcomes. Proteins represent essential macronutrients for the immune system. Amino acids are essential for synthesizing immune proteins, including antibodies and cytokines that mediate immune responses [8]. One of the crucial aspects of the impact of macronutrients on the immune system is taking part in immune distinction. Carbohydrates define cell surface molecules that can be identified as antigens by Toll-like receptors (TLRs) [9]. Fatty acids are essential energy sources, are components of cell membranes and modulate cell operation by acting as signaling molecules that regulate gene expression [10]. They can also affect the function of immune cells by being used as precursors for synthesizing lipid compounds involved in regulating immune responses and inflammatory pathways [10, 11].

Most studies have investigated the effect of the COVID-19 pandemic on nutritional intake, and according to our knowledge, no study has investigated the role of the intake of macronutrients in the incidence and severity of COVID-19 applying advanced mathematical approaches. Considering the importance of macronutrient intake in the immune system function, we aimed to investigate the effect of macronutrient intake on the incidence and severity of COVID-19 by dynamical system models. In terms of clinical implications, the results of this study may lead to dietary guidelines and policies which may prevent from such pathogenic infections in the future.

## Materials and methods

### Study design

**Fig. 1:**
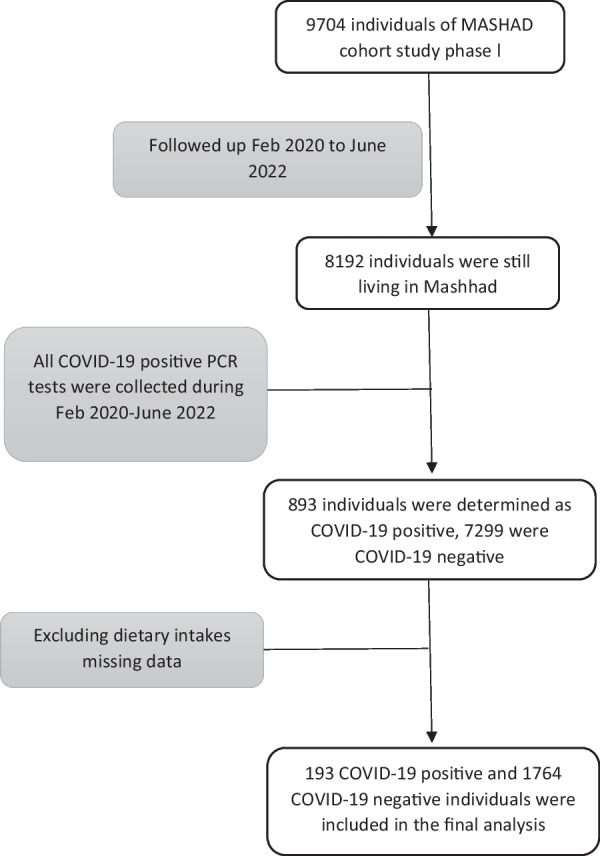
Flow diagram of study design

In this cohort study, the study population were recruited from Mashhad stroke and heart atherosclerotic disorder (MASHAD) study, a cohort of 9704 individuals aged 35–65 [12]. The MASHAD study started in 2007, and participants were followed-up after ten years. To determine the COVID-19 incidence, data of all PCR-positive patients from all related medical centers in Mashhad were recorded from February 2020 to June 2022 (*n* = 405,398). To determine the incidence of COVID-19 in the population of MASHAD study, we merged the COVID-19 data with the subjects in the first phase of MASHAD study, who were still living in Mashhad, based on the follow-up data of MASHHAD study. The MASHAD study population were 48–78 years at the time of COVID-19 pandemic. Among 8192 subjects, 893 were identified with a positive PCR test. After excluding the records with incomplete dietary intake data, 1957 were remained. Among the 1957 subjects, 193 were COVID-19-positive (Fig. 1). To determine the severity of COVID-19, hospital data were recorded based on the hospital information system (HIS).

### Dietary intake assessment

A 24-h dietary recall was applied to assess dietary intakes in the first phase of MASHAD study. An experienced nutritionist administered the questionnaire. Besides, the demographic characteristics were also recorded. Diet plan 6 software (Forestfield Software Ltd., Horsham, West Sussex, UK) was applied to analyze the energy and nutrient intakes. Macronutrient intakes were all adjusted for energy.

### Statistical analysis

Statistical analysis was performed using the statistical package for the Social Science version 25.0 (SPSS, Chicago, IL). The Kolmogorov–Smirnov test was used to check the normality of variables; normally distributed variables were described as mean ± SD. Not normally distributed variables were described as median and IQR (interquartile range). Multiple logistic regression was applied in IBM SPSS statistics 25.0. The dependent binary variables were COVID-19 incidence and hospitalization. Macronutrients (protein, Saturated fatty acids, monounsaturated fatty acid (MUFA), polyunsaturated fatty acid (PUFA), trans fatty acids, cholesterol, starch, total sugar, fiber, non-starch polysaccharides (NSP), and water were included in the model using a backward variable selection method, by adjusting for age and sex. To avoid multicolinearity in the regression model, the linear relationship of predictors was assessed. As NSP and fiber were correlated, we only add fiber to our model. There was no linear relationship among other variables. All analyses were two-sided, and *P*-value < 0.05 was considered statistically significant.

Applying MedCalc software, the best cut-off value was determined for COVID-19 incidence. The optimal cut-off values were defined as the point at which the value of sensitivity + specificity – 1 was maximum. This cut-off value corresponded to the point on the ROC curve with the maximum vertical distance from the curve to the chance line and has also been defined as an accuracy indicator in clinical epidemiology. Then, we considered a four-dimensional mathematical model based on the susceptible-exposed-infectious-removed (SEIR) model in the dynamics of mineral intake for COVID-19 patients. In the differential equations of SEIR, we consider people who enter the study as susceptible people. In these equations, patients are transferred from one group to another during division, and they decrease or increase with the percentage of transfer and the amount of consumed substances over time. Then, according to the dietary macronutrient intakes, we divided the susceptible individuals into two groups; we examined these groups of patients for COVID-19 infection and hospitalization.

Based on the population dynamics, differential equations were established. Then, by OD45 numerical methods using Matlab software, the model parameters were estimated using the second error powers and other error optimization methods. Finally, the statistical model was fitted to evaluate the results obtained.

### Ethical approval

All participants provided their written informed consent. The study protocol was approved by the Human Research Ethics Committee of Mashhad University of Medical Sciences (MUMS).

## Results

Baseline characteristics are presented in Table 1. The results showed no significant difference between subjects with and without COVID-19, except for NSP and fiber. Dietary intakes of NSP and fiber were significantly lower in COVID-19 patients (*P* < 0.05).

**Table 1:** Evaluation of macronutrient dietary intakes in patients with and without COVID-19

	COVID-19-positive *N* = 193	COVID-19-negative *N* = 1764	Effect size	*P* value
Age (year)	59 (56, 62)*	59 (57, 63)	0.08	0.12
Sex, *n* (%) Male Female	98 (50.78) 95 (49.22)	945 (53.57) 819 (46.43)	0.05	0.46
Protein (g/day)	69.08 (56.99, 79.47)	70.03 (60.87, 81.30)	0.001	0.19
Saturated fat (g/day)	17.015 (14.012, 20.77)	17.76 (14.40, 22.19)	0.002	0.059
MUFA (g/day)	18.924 ± 6.84**	19.89 ± 6.70	0.09	0.104
PUFA(g/day)	24.62 ± 13.18	24.02 ± 13.02	0.03	0.60
Trans fat (g/day)	1.712 ± 0.68	1.80 ± 0.65	0.014	0.14
Cholesterol (mg/day)	174.46 (118.48, 304.76)	187.8 (125.5, 303.2)	0.011	0.69
Starch (mg/day)	2786.9 ± 1111.19	2928 ± 964.68	0.023	0.132
Total sugar (mg/day)	79.27 (59.71, 102.88)	82.23 (59.44, 108.81)	0.012	0.99
NSP (g/day)	11.33 (7.48, 14.43)	11.88 (8.46, 15.86)	0.036	0.044
Fiber (g/day)	14.88 (10.09, 18.68)	15.49 (11.08, 21.10)	0.019	0.044
Water (mL/day)	1433.1 (1099.4, 1783.2)	1491.2 (1165.8, 1860.9)	0.011	0.37

*MUFA* Monounsaturated fatty acids, *NSP* non-starch polysaccharides, *PUFA* Polyunsaturated fatty acids

*Median (1st, 3st), **Mean ± SD

As shown in Table 2, PUFA, starch, total sugar, and fiber remained in the final model.

**Table 2:** Multiple logistic regression analysis for determination of the predictive variables for macronutrients in COVID-19

Variables	Crude	*P* value	Adjusted^a^	*P* value
	OR^b^ (95% CI^c^)		OR (95% CI)	
PUFA (g)	1.0048 (0.9919, 1.0179)	0.464	1.0023 (1.0005, 1.0040)	0.174
Starch (mg)	1.0030 (0.9995, 1.0066)	0.089	1.0008 (1.0003, 1.0012)*	0.001
Total sugar (mg)	1.0030 (0.9995, 1.0066)	0.089	1.0006 (1.0001, 1.0011)*	0.026
Fiber (g)	0.9856 (0.9645, 1.0072)	0.189	0.9985 (0.9965, 1.0005)	0.138

*PUFA* Polyunsaturated fatty acids

AIC = 631.9649, BIC = 662.8360

*P < 0.05 was considered statistically significant

^a^Adjusted for all of variables

^b^Odds ratio (OR)

^c^Confident interval (CI)

In the crude model, unadjusted for any confounding variable, there was no significant association between macronutrients intake and the odds of COVID-19 infection. After adjusting for age and sex, intakes of starch and total sugar were significantly associated with the increased odds of COVID-19 infection (*P* < 0.05).

Table 3 shows the macronutrients association with odds of hospitalization due to COVID-19. In the crude model, there was a significant association between fiber intake and hospitalization (*P* = 0.04) as each unit increase in fiber intake decreased odds of hospitalization by 10%. This association remained statistically significant after adjusting for confounding variables (*P* < 0.05).

**Table 3:** Multiple logistic regression analysis for determination of the predictive variables for macronutrients in COVID-19

Variables	Crude	*P* value	Adjusted^a^	*P* value
	OR^b^ (95% CI^c^)		OR (95% CI)	
Cholesterol (mg)	0.9960 (0.9905, 1.0015)	0.1562	1.0000 (0.9999, 1.0000)*	0.0779
Fiber (g)	0.9088 (0.8294, 0.9958)*	0.0404	0.9993 (0.9987, 0.9999)*	0.0187

AIC = 115.19, BIC = 135.77

*P < 0.05 was considered statistically significant

^a^Adjusted for all of variables

^b^Odds Ratio (OR)

^c^Confident Interval (CI)

### Dynamical system

The optimum cut-offs for macronutrient intake were determined using MedCalc software (Table 4). The best cut-off for cholesterol, fiber, PUFA, starch, and total sugar was 180.23 mg, 21.48 g, 13.11 g, 158.67 mg, and 79.53 mg, respectively.

**Table 4:** The best cut-offs for macronutrients intake using MedCalc software

Variable	Best cut-off points	Sensitivity	Specificity	Youden indexes	AUC (95% CI)
Cholesterol (mg)	180.23	49.93	52.87	0.0279	0.501 (0.489, 0.514)
Fiber (g)	21.48	27.61	74.84	0.0244	0.506 (0.492, 0.520)
PUFA (g)	13.11	18.46	84.59	0.0346	0.500 (0.487, 0.514)
Starch (mg)	158.67	64.23	38.77	0.0300	0.501 (0.488, 0.514)
Total sugar (mg)	79.53	49.38	53.59	0.0296	0.503 (0.489, 0.516)

*AUC* area under the curve, *PUFA* polyunsaturated fatty acids

To predict the effect of macronutrient intake on COVID-19, dynamics were modeled using a modified SEIR (susceptible-exposed-infected-removed) differential equation machine (Fig. 1). In this model, all participants are susceptible to COVID-19 (S). Participants who consumed the macronutrient more than the cut-off are presented as E_1_ and the group who consumed less than the mentioned cut-off are presented as E_2_. I_1_ is the group with a positive COVID-19 PCR, and I_2_ were not infected. If we consider all the participants in the modified equation model, we have the following equation:

$${\text{N}}({\text{t}}) = {\text{S}}({\text{t}}) + {\text{E}}_{1} ({\text{t}}) + {\text{E}}_{2} ({\text{t}}) + I_{1} ({\text{t}}) + {\text{I}}_{2} ({\text{t}}) + {\text{H}}({\text{t}}) + {\text{OU}}({\text{t}})$$

where COVID-19-positive patients who were hospitalized are represented with H. OU shows the patients who were managed in the outpatient setting. Considering the model above, the differential equation was solved using the numerical methods of OD45 in the Matlab software.

1 $$\left\{ {\begin{array}{*{20}l} {\begin{array}{*{20}c} {\begin{array}{*{20}c} {\begin{array}{*{20}c} {\dot{S} = - \left( {1 + {\text{m}}} \right)s + L} \\ {\mathop {{\text{E}}_{1} }\limits^{.} = - \left( {1 + {\text{m}}} \right){\text{E}}_{1} + \alpha s} \\ {\mathop {{\text{E}}_{2} }\limits^{.} = - \left( {\eta _{3} + {\text{m}}} \right){\text{E}}_{2} + (1 - \alpha )s} \\ \end{array} } \\ {\mathop {{\text{I}}_{1} }\limits^{.} = \gamma _{1} {\text{E}}_{1} + \gamma _{3} {\text{E}}_{2} - \left( {\beta _{1} + \beta _{2} + {\text{m}}_{1} } \right){\text{I}}_{1} } \\ \end{array} } \\ {\mathop {{\text{I}}_{2} }\limits^{.} = \gamma _{2} {\text{E}}_{1} + \gamma _{4} {\text{E}}_{2} - m{\text{I}}_{2} - \beta _{3} {\text{I}}_{2} } \\ {\dot{H} = \beta _{1} {\text{I}}_{1} - {\text{m}}_{1} H} \\ \end{array} } \hfill \\ {\begin{array}{*{20}c} {\mathop {{\text{OU}}}\limits^{.} = \beta _{2} {\text{I}}_{2} - {\text{m}}_{1} OU + \beta _{3} {\text{I}}_{2} } \\ {\dot{D} = {\text{m}}s + {\text{mE}}_{1} + {\text{mE}}_{2} + {\text{m}}_{1} {\text{I}}_{1} + {\text{m}}_{1} {\text{H}} + {\text{m}}I_{2} + {\text{m}}_{1} {\text{OU}}} \\ \end{array} } \hfill \\ \end{array} } \right.$$

**Fig. 2:**
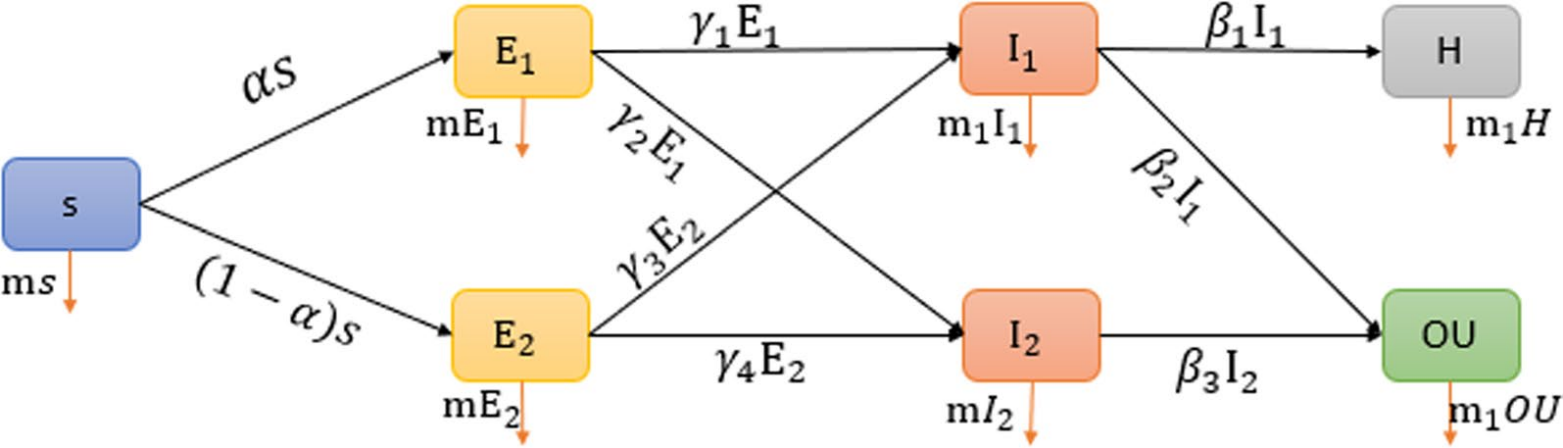
The modified SEIR (susceptible-exposed-infected-removed) model to predict COVID-19 infection and outcome. S: susceptible, E_1_: patients who consume the macronutrient above the cut-off, E_2_: patients who consume the macronutrient below the cut-off, I_1_: COVID-19-positive patients, I_2_: COVID-19-negative patients, H: hospitalized, OU: outpatient, α: Rate of transmission from S to E_1_, 1−α: Rate of transmission from S to E_2_, m: rate of death in susceptible individuals,${\gamma }_{1}$:Rate of transmission from E_1_ to I_1_,${\gamma }_{2}:$ Rate of transmission from E_1_ to I_2_, ${\gamma }_{3}$:Rate of transmission from E_2_ to I_1_, ${\gamma }_{4}:$ Rate of transmission from E_2_ to I_2_$, {\beta }_{1}:$ Rate of transmission from I_1_ to H, ${\beta }_{2}$: Rate of transmission from I_1_ to OU, ${\beta }_{3}$: Rate of transmission from I_2_ to OU

**Fig. 3:**
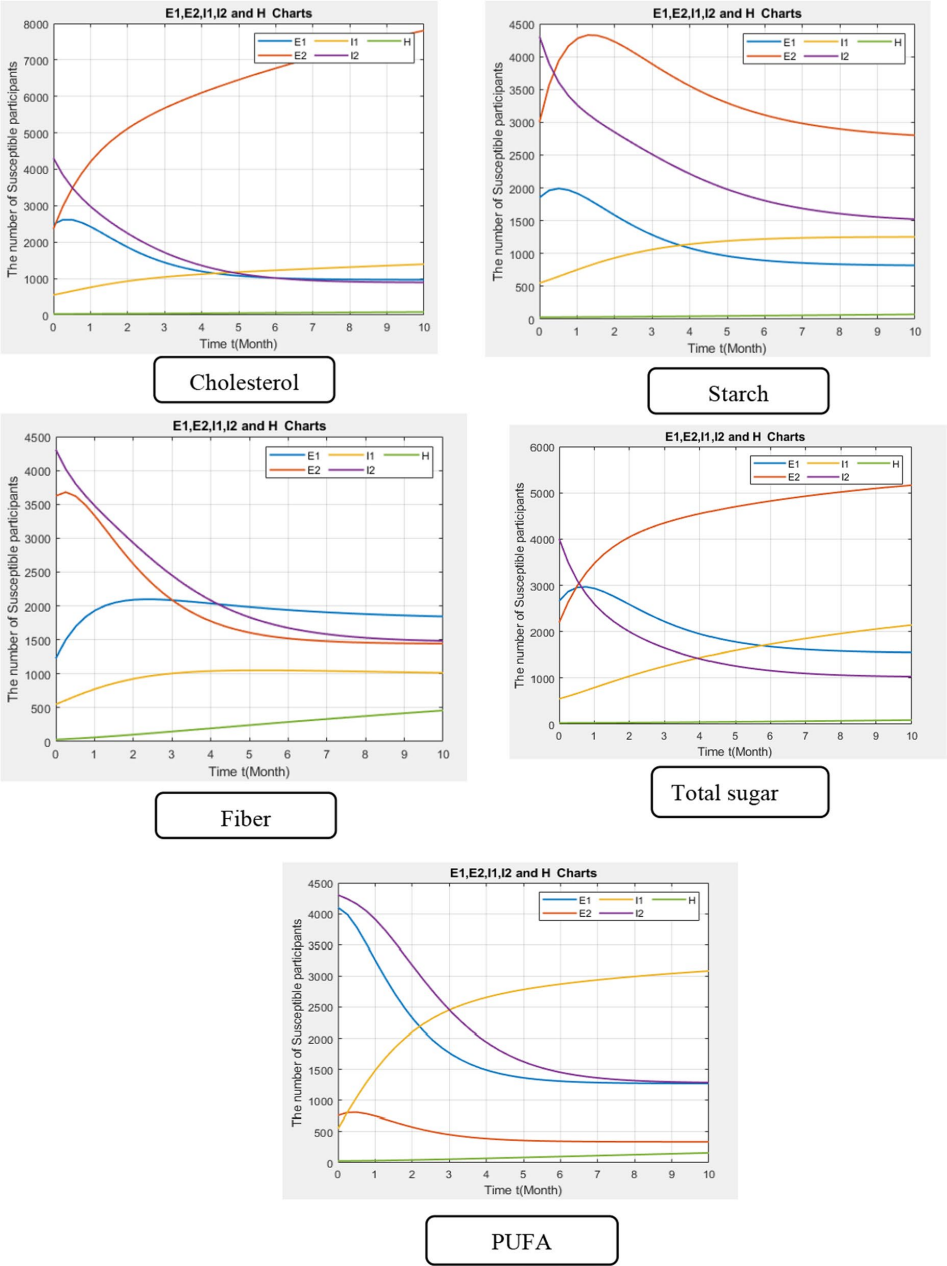
The model parameters for each macronutrient. E_1_: patients who consume the macronutrients above the cut-off, E_2_: patients who consume the macronutrients below the cut-off, I_1_: COVID-19-positive patients, I_2_: COVID-19-negative patients, H: hospitalized, PUFA: polyunsaturated fatty acids

The model parameters for each macronutrient are presented in Appendix 1. As illustrated in Fig. 2, the number of participants who consume cholesterol above the cut-off (E_1_) is decreasing, while the number of participants who use cholesterol below the cut-off (E_2_) is increasing. It shows that the increase in COVID-19-positive patients (I_1_) is more related to the group who use cholesterol above the cut-off. In fact, participants move from E_1_ to I_1_. Considering the diagram slopes, the risk of developing COVID-19 in the E_1_ group is 5.5 times greater than in the E_2_ group. The pattern of movements for total sugar is similar to cholesterol, as patients move from E_1_ to I_1_. Considering PUFA consumption, there is a sharp decrease in E_1_ and a nearly steady state in E_2_. The sharp decline in E_1_ justifies the sharp increase in I_2_. In fact, patients who consume PUFA above the cut-off are more susceptible to moving to the COVID-19-positive group. Considering fiber, the risk of COVID-19 in patients with a daily consumption of less than 21.48 g is 2.9 times greater than those with a daily fiber intake above the cu-toff (Fig. 3).

## Discussion

This study showed that dietary fiber intake in COVID-19-infected patients was significantly lower than in the non-COVID-19 group. It was also demonstrated that dietary intake of starch and total sugar was significantly associated with increased odds of COVID-19. On the other hand, fiber intake was significantly associated with decreased odds of COVID-19-related hospitalization. The results of the dynamical system analysis also revealed that participants who consumed cholesterol, total sugar, and PUFA above the calculated cut-off had a greater chance of future risk of COVID-19. At the same time, fiber intake above the cut-off showed a protective role.

Based on our results, dietary intake of total sugar was associated with increased odds of COVID-19. Carbohydrates with high glycemic index and subsequent acute insulin response lead to an overload of mitochondrial capacity and increased production of free radicals. Also, the consumption of simple carbohydrates causes an immediate increase in inflammatory cytokines and C-reactive protein [13]. Increased inflammatory responses and oxidative stress promote dysregulation in the innate immune system, resulting in an increased risk of infections [14].

According to the study results, dietary intake of fiber was significantly lower in COVID-19 patients, and each unit increase in dietary intake of fiber was associated with 1% decrease in the odds of hospitalization due to COVID-19. Dietary fibers are important factors regarding immunomodulation and decrease inflammation. A significant decrease in hs-CRP concentration has been reported with increasing fiber consumption [15]. Dietary fiber can also have a prebiotic effect by stimulating the growth of beneficial microbes such as Lactobacillus sp. and Bifidobacterium sp. and inhibiting pathogens such as *Clostridium* sp. [16]. In a study conducted by Hegazy et al. [17] on 200 patients with COVID-19, daily consumption of foods containing prebiotics, less sugar, regular exercise, adequate sleep and fewer antibiotic prescriptions led to a milder disease and faster clearance of the virus. Fiber sources and prebiotics indirectly affect the immune system by changing the composition and population of the gut microbiota [18]. In addition to changes in gut microbiota composition, prebiotics and most fibers can cause significant changes in the immune system by raising the expression of anti-inflammatory cytokines while decreasing the expression of pro-inflammatory cytokines [19, 20]. In an interventional study, Zhang et al. [21] investigated the effect of a new formula of bifidobacteria strains, galacto-oligosaccharides, xylo-oligosaccharides, and resistant dextrin (SIM01) in patients with COVID-19. They observed that patients receiving SIM01 developed significantly more SARS-CoV-2 IgG antibodies by day 16 than controls, and at week 5, plasma levels of IL-6, monocyte chemoattractant protein-1 (MCP-1), macrophage colony-stimulating factor (M-CSF), TNF-α, and IL-1RA significantly decreased in SIM01 but not in the control group.

The beneficial effects of prebiotics are believed to be mainly induced by increasing short-chain fatty acids (SCFA) production and strengthening the immune system of the gastrointestinal tract. It has also been found that prebiotics such as wheat bran, fructo-oligosaccharides, and galactosaccharides are known to raise butyrate levels, thereby decreasing inflammation and improving asthma and cystic fibrosis conditions [22, 23]. It is evident that dietary fiber-mediated modulation of gut microbiota and even lung microbiota can affect immunity and decrease the severity of viral infection in both the gastrointestinal and respiratory tracts [24]. Since fibers and prebiotics are generally safe, this microbiome therapy may improve and accelerate the recovery of patients with COVID-19, with less need for hospitalization.

Considering the dynamic system results, cholesterol consumption above the daily cut-off of 180 mg was associated with an increased risk of COVID-19. Cholesterol-rich food, often provided by unhealthy diets, affects the inflammatory markers and lipoprotein metabolism, modulating the lipoprotein profile. Based on animal and human studies, a high-cholesterol diet may increase the risk of pulmonary bacterial infections. Dietary cholesterol may also aggravate viral infections [25]. Our results also showed that patients who consume PUFAs above the cut-off are more susceptible to COVID-19. Among nutrients, omega-3 PUFAs are known to resolve inflammatory responses and support the immune system. In vivo and in vitro studies support the beneficial role of omega-3 PUFAs in lessening COVID-19 complications [26]. Conversely, eicosanoids from omega-6 (ω-6) PUFAs increase inflammatory responses [27]. Generally, it should be considered that excessive intake of PUFAs may be associated with dyslipidemia, dysregulation of glucose homeostasis, and immunosuppression [26].

This study had some limitations. First, we determined the COVID-19-positive patients based on PCR-positive results performed in all related clinical sites in Mashhad. We considered all other patients without a PCR-positive result as COVID-19-negative. It should be considered that some patients affected by SARS-COV2 are asymptomatic or may not perform a PCR test. Besides, our study has not considered the frequent infections with SARS-COV2 in individuals. Besides, although 24-h dietary recall can be used to assess dietary intakes of the population, it is not the ideal instrument to investigate the association of diet as an independent variable with health outcomes. Another limitation was that we excluded subjects with dietary missing data, which may affect the generalizability of our results. It should also be noted that some medical conditions such as diabetes or impaired glucose tolerance can be related to the risk of COVID-19 and its severity that have not been included in our analytical models.

During the COVID-19 pandemic, plenty of studies have been published on dietary changes due to COVID-19 and mechanistically or clinically reviewing the importance of nutrition in preventing or managing COVID-19. There are few studies concerning the effect of nutritional intake on COVID-19 susceptibility and severity. In this study, we evaluated the impact of previous dietary intakes on COVID-19 infection during the pandemic period. Another strength point of this study was that we applied advanced dynamical system analysis methods to predict the risk of COVID-19 in the future. Although COVID-19 is now subsiding in our region, the results of this study may help prevent and better manage the future new emerging infectious disease crisis such as COVID-19.

## Conclusion

Overall, this study showed that dietary fiber and NSP intake were lower in COVID-19 patients. Dietary intake of starch and total sugars were associated with increased odds of COVID-19, while fiber intake decreased the odds of hospitalization due to COVID-19. The dynamical system models showed that dietary cholesterol, PUFAs, and total sugar were associated with an increased risk of COVID-19, while fiber had a protective role.

## Data Availability

The datasets generated and/or analyzed during the current study are not publicly available due to university data ownership policies, but are available from the corresponding author on reasonable request.

## Appendix 1

See Tables 5 and 6.

**Table 5:** Description of variables in the dynamical system model

Variable	Interpretation	Cholesterol	Starch	Fiber	PUFA	Total sugar	Source
S	Macronutrient factors	5896	5759	4968	5190	4856	Data
E_1_	Macronutrient nutritional > cut-off in Medcalc	2492	1853	1229	4099	2662	Data
E_2_	Macronutrient nutritional $\le$ cut-off in Medcalc	2364	3003	3624	757	2194	Data
I_1_	COVID positive	550	550	550	550	550	Data
I_2_	COVID negative	4306	4306	4306	4303	4006	Data
H	Hospitalization	29	29	29	29	29	Data
OU	Outpatient	521	521	521	521	521	Data

S: susceptible, E_1_: patients who consume the macronutrient above the cut-off, E_2_: patients who consume the macronutrient below the cut-off, I_1_: COVID-19-positive patients, I_2_: COVID-19-negative patients, H: hospitalized, OU: outpatient, PUFA: polyunsaturated fatty acids

**Table 6:** Description of parameters in the dynamical system model

Parameters	Interpretation	Cholesterol	Starch	Fiber	PUFA	Total sugar	Source
$\mathrm{\alpha }$	Rate of transmission from S to E_1_	0.51	0.32	0.32	0.79	0.54	Data
$1-\mathrm{\alpha }$	Rate of transmission from S to E_2_	0.48	0.68	0.68	0.21	0.45	Data
$m$	The rate of death in susceptible individuals, E_1_, E_2_ and I_2_	0.045	0.045	0.045	0.045	0.045	Estimate
${\gamma }_{1}$	Rate of transmission from E_1_ to I_1_	0.094	0.07	0.031	0.13	0.06	Data
${\gamma }_{2}$	Rate of transmission from E_1_ to I_2_	0.74	0.54	0.22	0.86	0.48	Data
${\gamma }_{3}$	Rate of transmission from E_2_ to I_1_	0.0176	0.04	0.082	0.72	0.054	Data
${\gamma }_{4}$	Rate of transmission from E_2_ to I_2_	0.0137	0.33	0.66	0.27	0.0.39	Data
${\beta }_{1}$	Rate of transmission from I_1_ to H	0.006	0.005	0.05	0.006	0.005	Data
${\beta }_{2}$	Rate of transmission from I_1_ to OU	0.1069	0.106	0.107	0.09	0.106	Data
${\beta }_{3}$	Rate of transmission from I_2_ to OU	0.877	0.87	0.88	0.88	0.87	Data

α: Rate of transmission from S to E_1_, 1−α: Rate of transmission from S to E_2_, m: rate of death in susceptible individuals, ${\gamma }_{1}$: Rate of transmission from E_1_ to I_1_,${\gamma }_{2}:$ Rate of transmission from E_1_ to I_2_, ${\gamma }_{3}$: Rate of transmission from E_2_ to I_1_, ${\gamma }_{4}:$ Rate of transmission from E_2_ to I_2_,${\beta }_{1}:$ Rate of transmission from I_1_ to H, ${\beta }_{2}$: Rate of transmission from I_1_ to OU, ${\beta }_{3}:$ Rate of transmission from I_2_ to OU
